# New Urea and Thiourea Derivatives of Piperazine Doped with Febuxostat: Synthesis and Evaluation of Anti-TMV and Antimicrobial Activities

**DOI:** 10.1155/2013/682603

**Published:** 2013-12-26

**Authors:** Reddivari Chenna Krishna Reddy, Syed Rasheed, Devineni Subba Rao, Shaik Adam, Yellala Venkata Rami Reddy, Chamarthi Naga Raju

**Affiliations:** ^1^Department of Chemistry, Sri Venkateswara University, Tirupati, Andhra Pradesh-517 502, India; ^2^Department of Biochemistry, Sri Venkateswara University, Tirupati, Andhra Pradesh-517 502, India

## Abstract

A series of new 4-(5-(3-cyano-4-isobutoxyphenyl)-4-methylthiazole-2-carbonyl)-N-(substituted phenyl)piperazine-1-carboxamides **8(a–e)**/carbothioamides **8(f–j)** were accomplished for biological interest by the simple addition of active functionalized arylisocyanates **7(a–e)**/arylisothiocyanates **7(f–j)** with 2-isobutoxy-5-(4-methyl-2-(piperazine-1-carbonyl)thiazol-5-yl)benzonitrile **(4)**. Compound **4** was synthesized in high yields (94%) by the condensation reaction of febuxostat (1) with piperazine using a selective reagent such as propylphosphonic anhydride (T_3_P). Antiviral activity against *Tobacco mosaic virus* (TMV) and antimicrobial activity of the synthesized compounds were evaluated. Biological data revealed that 4-nitrophenyl substituted urea **8d**, and 3-bromophenyl substituted thiourea **8f** exhibited promising antiviral activities. Moreover, 4-fluorophenyl substituted urea **8a**, 4-nitrophenyl substituted urea **8d**, 3-bromophenyl substituted thiourea **8f**, and 2,4-dichlorophenyl substituted thiourea **8j** exhibited potent antimicrobial activity.

## 1. Introduction

**Figure 1:**
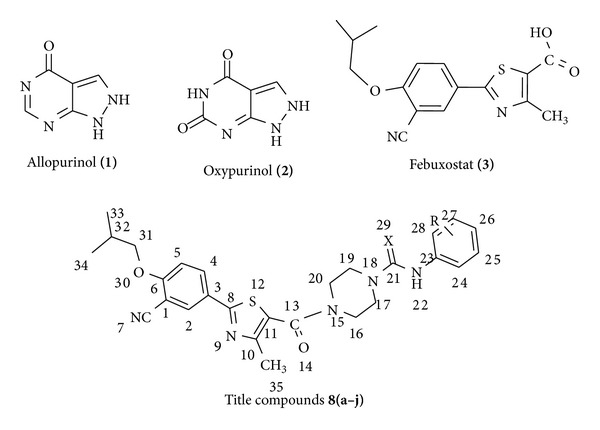
Some biologically active molecules.

Xanthine oxidase (XO) is an enzyme that catalyzes the oxidation of hypoxanthine to xanthine and can further catalyze the oxidation of xanthine to uric acid [1]. The literature studies revealed that an increase in mean serum urate in both genders during the past four decades [2–4] and the underlying cause of gout is hyperuricemia. In human, the substance which acts as inhibitor of xanthine oxidase reduces the production of uric acid and several medications that inhibit xanthine oxidase are indicated for treatment of hyperuricemia and related medical conditions including gout [5]. Two types of XO inhibitors are in use: purine analogues, such as allopurinol (**1**) and oxypurinol (**2**), have long been employed in main therapy for the treatment of chronic gout in many countries, and nonpurine analogues, such as febuxostat (**3**) (Figure 1). Febuxostat (Adenuric *▼*), 2-[3-cyano-4-(2-methylpropoxy)phenyl]-4-methylthiazole-5-carboxylic acid is a nonpurine selective inhibitor of xanthine oxidase (XO) used for the treatment of hyperuricemia and gout and was approved in the USA. It is not expected to inhibit other enzymes involved in purine, pyrimidine synthesis, and metabolism at therapeutic concentrations [6–8].

Furthermore, it is well known that a number of nitrogen containing heterocyclic compounds exhibited a wide variety of biological activities. Among them, piperazine derivatives have owed their importance in many medicinal molecules. Piperazine derivatives were originally used in veterinary medicines which combat parasitic infections in poultry, stimulants at low doses, and hallucinations at higher level doses [9]. In addition to the nitrogen functionalities such as urea and thiourea which have been found in broad spectrum of biological activities like antidiabetic [10], anti-inflammatory [11], antiviral [12], antibacterial, antifungal, herbicidal [13, 14], antituberculosis [15–18], and antidepressant activity on central nervous system [19] as well as purification agents for the effluent of organic and inorganic molecules in industrial, agricultural, and mining industries [20]. Recently, some of urea-based compounds act as kinase inhibitors and as novel therapeutics in cancer treatment [21] due to their unique binding mode and kinase inhibition profile. Having more importance in pharmacological applications of urea and thiourea derivatives, there is a possibility to design more efficient urea and thiourea derivatives as antiviral and antimicrobial agents.

Based on the importance of urea and thiourea and piperazine derivatives and in the part of our research programme, we designed and synthesized a series of novel 5-(3-cyano-4-isobutoxyphenyl)-4-methylthiazole-2-carbonyl)-N-(substituted phenyl)piperazine-1-carboxamide/carbothiomide derivatives with a hope to obtain good results in the screening biological studies against *Tobacco mosaic virus* and antimicrobial pathogens (bacterial and fungal strains).

## 2. Materials and Methods

### 2.1. Chemistry

All chemicals and reagents used for the synthesis were commercially available, and AR grade solvents/reagents were used as such were received from Sigma-Aldrich and Merck. All solvents used for spectroscopic and other physical studies were reagent grade and were further purified by the literature methods [22]. All melting points (m.p) were obtained with a digital Guna melting point apparatus and are uncorrected. IR spectra were recorded on a Perkin Elmer 283 unit using KBr discs. ^1^H/^13^C NMR spectra were recorded on a Bruker 400 MHz NMR spectrometer operating at 400 MHz for ^1^H and 100.25 MHz for ^13^C in DMSO-*d*_6_ solvent and TMS was used as internal standard. Chemical shift values (*δ*) and coupling constants (*J*) are reported in ppm and Hz, respectively. Standard abbreviations indicating multiplicity are used as follows: “s” for singlet, “d” for doublet, “dd” for doublet of doublets, “t” for triplet, “q” for quartet, “m” for multiplet, and “br” for broad. LC-MS spectra were recorded on a Jeol SX 102 DA/600 Mass spectrometer. Elemental analyses were performed on a Thermo Finnigan Instrument at the University of Hyderabad, Hyderabad, India. Analytical thin-layer chromatography (TLC) was carried out on precoated plates and spots were visualized with UV light.

#### 2.1.1. Synthetic Procedure for the Synthesis of 2-Isobutoxy-5-(4-methyl-2-(piperazine-1-carbonyl)thiazol-5-yl)benzonitrile (**4**)

Febuxostat (**3**) (2.97 g, 9.4 mmol) was dissolved in 50 mL of dry dichloromethane (DCM) in a flask and base and diisopropylethylamine (DIPEA) (2.5 mL, 14.0 mmol) was added. The reaction mixture was stirred for 20 min at room temperature to get homogeneous solution. The reagent, T_3_P (3.6 mL, 12.0 mmol) and piperazine (0.81 g, 9.4 mmol) were added to the reaction mixture with stirring. Then, the reaction mixture was stirred vigorously for 8 h at room temperature. After completion of the reaction as checked by TLC, the solid was filtered off and recrystallized from methanol to afford 2-isobutoxy-5-(4-methyl-2-(piperazine-1-carbonyl)thiazol-5-yl)benzonitrile (**4**) (3.54 g) (94%). Mol. Wt 384, mp 154–156°C. IR (KBr, *υ* cm^−1^): 3334 (–N–H, str), 3015 (=C–H, str), 2240 (–CN, str), 1654 (–C=O, str). ^1^H NMR (DMSO-*d*_6_, 400 MHz) *δ* (ppm): 0.92 (d, 6H, *J* = 7.6 Hz, (CH_3_)_2_–CH–), 1.28–1.42 (m, 1H, (CH_3_)_2_–CH–CH_2_–), 2.43 (s, 3H, –CH_3_), 3.12 (t, 4H, *J* = 6.8 Hz, –CH_2_–N–CH_2_–), 3.28 (t, 4H, *J* = 6.8 Hz, –CH_2_–N–CH_2_–), 3.79 (d, 2H, *J* = 7.6 Hz, –O–CH_2_–CH–), 6.98 (d, 1H, *J* = 6.4 Hz, Ar-H), 7.21 (s, 1H, Ar-H), 7.64 (d, 1H, *J* = 6.0 Hz, Ar-H). LC-MS (*m/z*, %): 383 (M-H^+^, 100).

#### 2.1.2. Typical Procedure for the Synthesis of Title Urea/Thiourea Derivatives  **8**(**a**–**j**)

The synthesized intermediate, 2-isobutoxy-5-(4-methyl-2-(piperazine-1-carbonyl)thiazol-5-yl)benzonitrile (**4**) (250 mg, 0.65 mmol), 1-isocyanato-4-nitrobenzene (**7d**) (106.38 mg, 0.65 mmol) and N,N-dimethylpiperazine (0.13 mL, 0.98 mmol) were dissolved in 20 mL of THF in a flask and refluxed with vigorous stirring for 2.5 h. After completion of the reaction as indicated by TLC, the reaction mixture was concentrated under reduced pressure to get crude product **8d**. The crude product was purified by column chromatography using 3 : 7 ratio of ethyl acetate and *n*-hexane as an eluent to get pure 4-(5-(3-cyano-4-isobutoxyphenyl)-4-methylthiazole-2-carbonyl)-N-(4-nitrophenyl)piperazine-1-carboxamide (**8d**) (321 mg, 90%). All the other title compounds were synthesized using the above experimental procedure.

#### 2.1.3. 4-[2-(3-Cyano-4-isobutoxyphenyl)-4-methylthiazole-5-carbonyl]-N-(4-fluorophenyl)piperazine-1-carboxamide (**8a**)

Light brown solid, Yield: 85%, mp 182–184°C. IR (KBr, *υ* cm^−1^): 3296 (–N–H, str), 3015 (=C–H, str), 2889 (–C–H, str), 2243 (–CN, str), 1676 (–C=O, str), 1638 (–C=O, str), 1167 (–C–F, str); ^1^H NMR (DMSO-*d*_6_, 400 MHz) *δ* (ppm): 0.94 (d, 6H, *J* = 8.8 Hz, (CH_3_)_2_–CH–), 1.28–1.42 (m, 1H, (CH_3_)_2_–CH–CH_2_–), 2.56 (s, 3H, –CH_3_), 3.39 (t, 4H, *J* = 7.2 Hz, –CH_2_–N–CH_2_–), 3.68 (t, 4H, *J* = 6.8 Hz, –CH_2_–N–CH_2_–), 3.85 (d, 2H, *J* = 7.6 Hz, –O–CH_2_–CH–), 7.36–7.43 (m, 2H, Ar-H), 7.61 (d, 2H, *J* = 6.4 Hz, Ar-H), 7.73–7.81 (m, 3H, Ar-H), 9.51 (s, 1H, –NH–C=O); ^13^C NMR (DMSO-*d*_6_, 100.25 MHz) *δ* (ppm): 17.4 (C_35_), 18.9 (C_33,34_), 31.2 (C_32_), 48.6 (C_16,20_), 51.2 (C_17,19_), 73.1 (C_31_), 104.6 (C_1_), 113.9 (C_25,27_), 114.6 (C_5_), 118.2 (C_11_), 119.4 (C_7_), 120.1 (C_24,28_), 123.4 (C_3_), 126.2 (C_2_), 130.4 (C_4_), 136.6 (C_23_), 151.3 (C_6_), 154.8 (C_10_), 158.3 (C_21_), 162.2 (C_13_), 165.7 (C_26_), 166.2 (C_8_); LC-MS (*m/z*, %): 520 (M-H^+^, 100), 410 (M-H^+^ − 110, 30), 382 (M-H^+^ − 138, 73); Anal. Calcd. for C_27_H_28_FN_5_O_3_S: C, 62.17; H, 5.41; N, 13.43. Found: C, 61.93; H, 5.34; N, 13.48%.

#### 2.1.4. N-(4-Bromophenyl)-4-[2-(3-cyano-4-isobutoxyphenyl)-4-methylthiazole-5-carbonyl]piperazine-1-carboxamide (**8b**)

White solid, Yield: 81%, mp 163-165°C. IR (KBr, *υ* cm^−1^): 3312 (–N–H, str), 3018 (=C–H, str), 2885 (–C–H, str), 2235 (–CN, str), 1672 (–C=O, str), 1646 (–C=O, str), 748 (–C–Br, str); ^1^H NMR (DMSO-*d*_6_, 400 MHz) *δ* (ppm): 0.89 (d, 6H, *J* = 7.6 Hz, (CH_3_)_2_–CH–), 1.21–1.33 (m, 1H, (CH_3_)_2_–CH–CH_2_–), 2.51 (s, 3H, –CH_3_), 3.34 (t, 4H, *J* = 7.2 Hz, –CH_2_–N–CH_2_–), 3.51 (t, 4H, *J* = 7.2 Hz, –CH_2_–N–CH_2_–), 3.74 (d, 2H, *J* = 6.8 Hz, –O–CH_2_–CH–), 7.27 (d, 1H, *J* = 6.8 Hz, Ar-H), 7.18 (d, 2H, *J* = 6.4 Hz, Ar-H), 7.26–7.39 (m, 4H, Ar-H), 9.28 (s, 1H, –NH–C=O); ^13^C NMR (DMSO-*d*_6_, 100.25 MHz) *δ* (ppm): 17.1 (C_35_), 17.9 (C_33,34_), 33.4 (C_32_), 49.8 (C_16,20_), 51.4 (C_17,19_), 72.8 (C_31_), 104.4 (C_1_), 113.6 (C_25,27_), 114.8 (C_5_), 117.5 (C_11_), 118.1 (C_7_), 121.4 (C_24,28_), 122.7 (C_26_), 124.5 (C_3_), 125.8 (C_2_), 128.7 (C_4_), 136.3 (C_23_), 152.6 (C_6_), 154.7 (C_10_), 157.2 (C_21_), 160.1 (C_13_), 164.7 (C_8_); LC-MS (*m/z*, %): 582 (M-H^+^+2, 97), 580 (M-H^+^, 100), 410 (M-H^+^ − 170, 46).

#### 2.1.5. 4-[2-(3-Cyano-4-isobutoxyphenyl)-4-methylthiazole-5-carbonyl]-N-(2-nitrophenyl)piperazine-1-carboxamide (**8c**)

Brown powdered solid, Yield: 89%, mp 176–178°C. IR (KBr, *υ* cm^−1^): 3320 (–N–H, str), 3051 (=C–H, str), 2892 (–C–H, str), 2248 (–CN, str), 1674 (–C=O, str), 1640 (–C=O, str), 1546 (–NO_2_ (aromatic), asymstr); ^1^H NMR (DMSO-*d*_6_, 400 MHz) *δ* (ppm): 0.98 (d, 6H, *J* = 7.2 Hz, (CH_3_)_2_–CH–), 1.24–1.31 (m, 1H, (CH_3_)_2_–CH–CH_2_–), 2.73 (s, 3H, –CH_3_), 3.55 (t, 4H, *J* = 6.8 Hz, –CH_2_–N–CH_2_–), 3.68 (t, 4H, *J* = 7.2 Hz, –CH_2_–N–CH_2_–), 3.96 (d, 2H, *J* = 7.6 Hz, –O–CH_2_–CH–), 7.35 (d, 1H, *J* = 6.4 Hz, Ar-H), 7.64–7.79 (m, 4H, Ar-H), 8.18–8.26 (m, 2H, Ar-H), 9.88 (s, 1H, –NH–C=O); ^13^C NMR (DMSO-*d*_6_, 100.25 MHz) *δ* (ppm): 17.8 (C_35_), 17.9 (C_33,34_), 33.7 (C_32_), 50.1 (C_16,20_), 58.2 (C_17,19_), 73.6 (C_31_), 105.3 (C_1_), 115.4 (C_5_), 116.5 (C_11_), 120.8 (C_7_), 122.7 (C_28_), 124.6 (C_26_), 125.7 (C_25,3_), 129.8 (C_27_), 131.7 (C_2,4_), 135.6 (C_23_), 143.5 (C_24_), 152.1 (C_6_), 155.3 (C_10_), 156.8 (C_21_), 160.3 (C_13_), 164.3 (C_8_); LC-MS (*m/z*, %): 547 (M-H^+^, 100), 410 (M-H^+^ − 137, 38), 382 (M-H^+^ − 165, 71).

#### 2.1.6. 4-[2-(3-Cyano-4-isobutoxyphenyl)-4-methylthiazole-5-carbonyl]-N-(4-nitrophenyl)piperazine-1-carboxamide (**8d**)

Yellow solid, Yield: 90%, mp 181–183°C. IR (KBr, *υ* cm^−1^): 3315 (–N–H, str), 3042 (=C–H, str), 2896 (–C–H, str), 2257 (–CN, str), 1678 (–C=O, str), 1635 (–C=O, str), 1542 (–NO_2_ (aromatic), asymstr); ^1^H NMR (DMSO-*d*_6_, 400 MHz) *δ* (ppm): 1.04 (d, 6H, *J* = 7.2 Hz, (CH_3_)_2_–CH–), 1.19–1.28 (m, 1H, (CH_3_)_2_–CH–CH_2_–), 2.67 (s, 3H, –CH_3_), 3.42 (t, 4H, *J* = 7.2 Hz, –CH_2_–N–CH_2_–), 3.68 (t, 4H, *J* = 7.2 Hz, –CH_2_–N–CH_2_–), 3.92 (d, 2H, *J* = 6.8 Hz, –O–CH_2_–CH–), 7.35 (d, 1H, *J* = 6.4 Hz, Ar-H), 7.54–7.68 (m, 4H, Ar-H), 8.24 (d, 2H, *J* = 6.8 Hz, Ar-H), 10.05 (s, 1H, –NH–C=O); ^13^C NMR (DMSO-*d*_6_, 100.25 MHz) *δ* (ppm): 17.5 (C_35_), 17.9 (C_33,34_), 34.1 (C_32_), 49.5 (C_16,20_), 55.6 (C_17,19_), 71.9 (C_31_), 106.2 (C_1_), 116.9 (C_5_), 117.6 (C_11_), 120.3 (C_7_), 122.7 (C_24,28_), 125.4 (C_25,27_), 125.9 (C_3_), 132.4 (C_2,4_), 141.6 (C_26_), 145.8 (C_23_), 150.3 (C_6_), 155.4 (C_10_), 156.7 (C_21_), 159.4 (C_13_), 165.1 (C_8_); LC-MS (*m/z*, %): 547 (M-H^+^, 100), 382 (M-H^+^ − 165, 71). Anal. Calcd. for C_27_H_28_N_6_O_5_S: C, 59.11; H, 5.14; N, 15.32. Found: C, 58.96; H, 5.03; N, 15.20%.

#### 2.1.7. N-(3-Chloro-4-fluorophenyl)-4-[2-(3-cyano-4-isobutoxyphenyl)-4-methylthiazole-5-carbonyl]piperazine-1-carboxamide (**8e**)

White solid, Yield: 86%, mp 201–203°C. IR (KBr, *υ* cm^−1^): 3337 (–N–H, str), 3025 (=C–H, str), 2887 (–C–H, str), 2248 (–CN, str), 1675 (–C=O, str), 1630 (–C=O, str), 1154 (–C–F, str), 823 (–C–Cl, str); ^1^H NMR (DMSO-*d*_6_, 400 MHz) *δ* (ppm): 0.91 (d, 6H, *J* = 7.2 Hz, (CH_3_)_2_–CH–), 1.01–1.12 (m, 1H, (CH_3_)_2_–CH–CH_2_–), 2.49 (s, 3H, –CH_3_), 3.57 (t, 4H, *J* = 6.8 Hz, –CH_2_–N–CH_2_–), 3.61 (t, 4H, *J* = 6.4 Hz, –CH_2_–N–CH_2_–), 3.91 (d, 2H, *J* = 6.4 Hz, –O–CH_2_–CH–), 7.29–7.38 (m, 3H, Ar-H), 7.52 (s, 1H, Ar-H), 7.63–7.72 (m, 2H, Ar-H), 9.15 (s, 1H, –NH–C=O); ^13^C NMR (DMSO-*d*_6_, 100.25 MHz) *δ* (ppm): 17.9 (C_35_), 18.2 (C_33,34_), 34.5 (C_32_), 48.7 (C_16,20_), 54.8 (C_17,19_), 71.2 (C_31_), 105.4 (C_1_), 115.3 (C_5_), 116.1 (C_11_), 117.3 (C_27_), 118.8 (C_7_), 123.4 (C_24,28_), 123.9 (C_25_), 126.3 (C_3_), 131.1 (C_2,4_), 135.2 (C_23_), 152.1 (C_6_), 154.3 (C_10_), 154.8 (C_26_), 158.9 (C_21_), 160.8 (C_13_), 163.7 (C_8_); LC-MS (*m/z*, %): 556 (M-H^+^+2, 33), 554 (M-H^+^, 100), 382 (M-H^+^ − 172, 64).

#### 2.1.8. N-(3-Bromophenyl)-4-[2-(3-cyano-4-isobutoxyphenyl)-4-methylthiazole-5-carbonyl]piperazine-1-carbothioamide (**8f**)

White solid, Yield: 83%, mp 167–169°C. IR (KBr, *υ* cm^−1^): 3287 (–N–H, str), 3015 (=C–H, str), 2881 (–C–H, str), 2232 (–CN, str), 1673 (–C=O, str), 1187 (–C=S, str), 754 (–C–Br, str); ^1^H NMR (DMSO-*d*_6_, 400 MHz) *δ* (ppm): 0.93 (d, 6H, *J* = 7.2 Hz, (CH_3_)_2_–CH–), 1.32–1.38 (m, 1H, (CH_3_)_2_–CH–CH_2_–), 2.43 (s, 3H, –CH_3_), 3.61 (t, 4H, *J* = 7.6 Hz, –CH_2_–N–CH_2_–), 3.75 (t, 4H, *J* = 7.2 Hz, –CH_2_–N–CH_2_–), 3.84 (d, 2H, *J* = 6.8 Hz, –O–CH_2_–CH–), 6.47–6.53 (m, 4H, Ar-H), 7.35 (d, 1H, *J* = 6.4 Hz, Ar-H), 7.61–7.72 (m, 2H, Ar-H), 9.76 (s, 1H, –NH–C=O); ^13^C NMR (DMSO-*d*_6_, 100.25 MHz) *δ* (ppm): 18.4 (C_35_), 18.5 (C_33,34_), 32.1 (C_32_), 47.3 (C_16,20_), 51.4 (C_17,19_), 72.8 (C_31_), 103.2 (C_1_), 115.6 (C_5_), 115.8 (C_11_), 116.2 (C_7_), 124.1 (C_25_), 124.8 (C_24,28_), 125.7 (C_3_), 126.4 (C_26_), 129.3 (C_27_), 133.5 (C_2,4_), 137.7 (C_23_), 158.9 (C_6_), 159.2 (C_10_), 163.4 (C_13_), 166.4 (C_8_), 175.1 (C_21_); LC-MS (*m/z*, %): 598 (M-H^+^+2, 97), 596 (M-H^+^, 100), 382 (M-H^+^ − 214, 58).

#### 2.1.9. 4-[2-(3-Cyano-4-isobutoxyphenyl)-4-methylthiazole-5-carbonyl]-N-(4-nitrophenyl)piperazine-1-carbothioamide (**8g**)

Brown solid, Yield: 86%, mp 171–173°C. IR (KBr, *υ* cm^−1^): 3293 (–N–H, str), 3022 (=C–H, str), 2887 (–C–H, str), 2235 (–CN, str), 1678 (–C=O, str), 1536 (–NO_2_ (aromatic), asymstr), 1195 (–C=S, str); ^1^H NMR (DMSO-*d*_6_, 400 MHz) *δ* (ppm): 0.92 (d, 6H, *J* = 6.8 Hz, (CH_3_)_2_–CH–), 1.11–1.19 (m, 1H, (CH_3_)_2_–CH–CH_2_–), 2.78 (s, 3H, –CH_3_), 3.59 (t, 4H, *J* = 7.2 Hz, –CH_2_–N–CH_2_–), 3.82 (t, 4H, *J* = 7.2 Hz, –CH_2_–N–CH_2_–), 3.91 (d, 2H, *J* = 6.4 Hz, –O–CH_2_–CH–), 7.28 (s, 1H, Ar-H), 7.38 (d, 1H, *J* = 6.8 Hz, Ar-H), 7.61–7.71 (m, 3H, Ar-H), 8.22 (d, 2H, *J* = 6.0 Hz, Ar-H), 9.61 (s, 1H, –NH–C=O); ^13^C NMR (DMSO-*d*_6_, 100.25 MHz) *δ* (ppm): 18.7 (C_35_), 18.8 (C_33,34_), 33.6 (C_32_), 45.1 (C_16,20_), 50.8 (C_17,19_), 72.4 (C_31_), 103.4 (C_1_), 114.7 (C_5_), 115.1 (C_11_), 116.8 (C_7_), 124.3 (C_25,27_), 124.6 (C_24,28_), 125.0 (C_3_), 132.5 (C_2,4_), 141.5 (C_23_), 145.2 (C_26_), 160.2 (C_6_), 160.3 (C_10_), 164.5 (C_13_), 166.7 (C_8_), 177.4 (C_21_); LC-MS (*m/z*, %): 563 (M-H^+^, 100), 382 (M-H^+^ − 181, 66). Anal. Calcd. for C_27_H_28_N_6_O_4_S_2_: C, 57.43; H, 5.00; N, 14.88. Found: C, 57.36; H, 4.94; N, 14.85%.

#### 2.1.10. N-(4-Chlorophenyl)-4-[2-(3-cyano-4-isobutoxyphenyl)-4-methylthiazole-5-carbonyl]piperazine-1-carbothioamide (**8h**)

Light orange solid, Yield: 83%, mp 188–190°C. IR (KBr, *υ* cm^−1^): 3282 (–N–H, str), 3021 (=C–H, str), 2884 (–C–H, str), 2236 (–CN, str), 1674 (–C=O, str), 1182 (–C=S, str), 794 (–C–Cl, str); ^1^H NMR (DMSO-*d*_6_, 400 MHz) *δ* (ppm): 0.96 (d, 6H, *J* = 7.2 Hz, (CH_3_)_2_–CH–), 1.05–1.11 (m, 1H, (CH_3_)_2_–CH–CH_2_–), 2.75 (s, 3H, –CH_3_), 3.54 (t, 4H, *J* = 7.2 Hz, –CH_2_–N–CH_2_–), 3.80 (t, 4H, *J* = 7.2 Hz, –CH_2_–N–CH_2_–), 3.92 (d, 2H, *J* = 6.8 Hz, –O–CH_2_–CH–), 6.88 (d, 2H, *J* = 6.8 Hz, Ar-H), 7.27–7.41 (m, 4H, Ar-H), 7.64 (d, 1H, *J* = 6.4 Hz, Ar-H), 9.52 (s, 1H, –NH–C=O); ^13^C NMR (DMSO-*d*_6_, 100.25 MHz) *δ* (ppm): 18.1 (C_35_), 18.4 (C_33,34_), 32.7 (C_32_), 45.0 (C_16,20_), 52.4 (C_17,19_), 72.1 (C_31_), 103.8 (C_1_), 114.9 (C_5_), 116.7 (C_11_), 116.8 (C_7_), 124.3 (C_3_), 129.7 (C_25,27_), 130.3 (C_24,28_), 132.2 (C_2,4_), 134.5 (C_26_), 136.7 (C_23_), 159.6 (C_6_), 160.5 (C_10_), 163.7 (C_13_), 166.2 (C_8_), 175.3 (C_21_); LC-MS (*m/z*, %): 554 (M-H^+^+2, 33), 552 (M-H^+^, 100).

#### 2.1.11. 4-[2-(3-Cyano-4-isobutoxyphenyl)-4-methylthiazole-5-carbonyl]-N-(2,6-difluorophenyl)piperazine-1-carbothioamide (**8i**)

White solid, Yield: 83%, mp 214–216°C. IR (KBr, *υ* cm^−1^): 3285 (–N–H, str), 3024 (=C–H, str), 2892 (–C–H, str), 2241 (–CN, str), 1680 (–C=O, str), 1190 (–C=S, str), 1178 (–C–F, str); ^1^H NMR (DMSO-*d*_6_, 400 MHz) *δ* (ppm): 0.96 (d, 6H, *J* = 7.6 Hz, (CH_3_)_2_–CH–), 1.09–1.17 (m, 1H, (CH_3_)_2_–CH–CH_2_–), 2.78 (s, 3H, –CH_3_), 3.62 (t, 4H, *J* = 6.8 Hz, –CH_2_–N–CH_2_–), 3.73 (t, 4H, *J* = 6.8 Hz, –CH_2_–N–CH_2_–), 3.95 (d, 2H, *J* = 6.8 Hz, –O–CH_2_–CH–), 6.66–6.74 (m, 3H, Ar-H), 7.26 (d, 1H, *J* = 6.0 Hz, Ar-H), 7.46–7.58 (m, 2H, Ar-H), 10.69 (s, 1H, –NH–C=O); ^13^C NMR (DMSO-*d*_6_, 100.25 MHz) *δ* (ppm): 18.6 (C_35_), 19.1 (C_33,34_), 33.4 (C_32_), 45.8 (C_16,20_), 52.9 (C_17,19_), 72.5 (C_31_), 105.3 (C_1_), 113.8 (C_25,27_), 115.2 (C_5_), 115.4 (C_23_), 116.3 (C_11_), 117.2 (C_7_), 124.8 (C_3_), 126.4 (C_26_), 133.7 (C_2,4_), 159.7 (C_6_), 162.3 (C_10_), 164.5 (C_13_), 165.5 (C_24,28_), 166.7 (C_8_), 179.6 (C_21_); LC-MS (*m/z*, %): 554 (M-H^+^, 100).

#### 2.1.12. 4-[2-(3-Cyano-4-isobutoxyphenyl)-4-methylthiazole-5-carbonyl]-N-(2,4-dichlorophenyl)piperazine-1-carbothioamide (**8j**)

Light yellow solid, Yield: 82%, mp 142–144°C. IR (KBr, *υ* cm^−1^): 3267 (–N–H, str), 3016 (=C–H, str), 2886 (–C–H, str), 2248 (–CN, str), 1683 (–C=O, str), 1186 (–C=S, str), 823 (–C–Cl, str). ^1^H NMR (DMSO-*d*_6_, 400 MHz) *δ* (ppm): 0.94 (d, 6H, *J* = 7.2 Hz, (CH_3_)_2_–CH–), 1.09–1.14 (m, 1H, (CH_3_)_2_–CH–CH_2_–), 2.70 (s, 3H, –CH_3_), 3.57 (t, 4H, *J* = 7.2 Hz, –CH_2_–N–CH_2_–), 3.68 (t, 4H, *J* = 6.8 Hz, –CH_2_–N–CH_2_–), 3.91 (d, 2H, *J* = 6.8 Hz, –O–CH_2_–CH–), 7.34–7.56 (m, 4H, Ar-H), 7.86 (d, 1H, *J* = 6.0 Hz, Ar-H), 8.24 (s, 1H, Ar-H), 10.53 (s, 1H, –NH–C=O); LC-MS (*m/z*, %): 590 (M-H^+^+4, 11), 588 (M-H^+^+2, 66), 586 (M-H^+^,100).

### 2.2. Biological Activity

#### 2.2.1. Anti-TMV Activity

*(1) Purification of the Virus*. *Tobacco mosaic virus* (TMV) is one of the most studied plant virus and it is a major challenge for researchers to control it since it represents a major threat in the production of the cash crops like tobacco. Based on the literature reports on the thiourea and urea derivatives antiviral activity [12], the synthesized compounds **8(a**–**j**) were screened for their antiviral activity against TMV.

Anti-TMV activity was evaluated by using Gooding's method [23]. The upper leaves of *Nicotiana tabacum* L. were inoculated with *tobacco mosaic virus,* selected and ground in phosphate buffer, and then filtered through a double-layer pledget. The filtrate was centrifuged at 10000 g bybeing treated twice with polyethylene glycol (PEG) and centrifuged again. The whole experiment was carried out at 4°C. Absorbance of sample was measured at 260 nm using ultraviolet spectrophotometer. The concentration of virus was calculated by using the following formula:

(1) $$\begin{array}{c} \text{Virus}\;\text{concn}=\frac{\left({\text{A}}_{260}\times \text{dilution}\;\text{ratio}\right)}{E_{1\;\text{cm}}^{0.1\%,\;260\;\text{nm}}}. \end{array}$$

*(2) In Vivo Antiviral Activity*

*Protective Effect*. The compound solutions were smeared on the left side, whereas the solvent served as the control on the right side of the growing *N. tabacum* L. leaves of the same age. The leaves were then inoculated with the virus of 6 × 10^−3^ mg/mL after 12 h, which were previously scattered with silicon carbide. The leaves were then washed with water and rubbed softly along the nervature twice. The local lesions appearing after inoculation of 3-4 days were counted. The inhibition rate of the tested samples was calculated according to the following formula. The experiments were repeated in triplicated and average values were taken as a final result as follows:

(2) $$\begin{array}{c} \text{Inhibition}\;\text{rate}\;\%=\left[\frac{x-y}{x}\right]\times 100. \end{array}$$

Here, *x* = av is the number of local lesions in control, *y* = av is the number of local lesions in tested sample. (av denotes average and control was not treated with compounds).

*Inactivation Effect*. The virus was inhibited by mixing with the compound solutions at the same volume for 30 min. The mixture was then inoculated on the left side of the *N. tabacum* L. leaves and the right side of the leaves was inoculated with the mixture of solvent and the virus alone. The leaves were then washed with water and rubbed softly along the nervature twice. The local lesions appearing after inoculation of 3-4 days were counted. The inhibition rate of the tested samples was calculated according to the following formula. The experiments were repeated in triplicated and average values were taken as a final result as follows:

(3) $$\begin{array}{c} \text{Inhibition}\;\text{rate}\;\%=\left[\frac{x-y}{x}\right]\times 100. \end{array}$$

Here, *x* = av is the number of local lesions in control, *y* = av no. is the number of local lesions in tested sample. (av denotes average and control was not treated with compounds).

*Curative Effect*. The same aged growing *N. tabacum* L. leaves were selected. The concentration of TMV, 6 × 10^−3^ mg/mL was dipped and inoculated on the whole leaves. Then, the leaves were washed with water and air dried for a few seconds. The compound solution was smeared on the left side and the solvent was smeared on the right side for control. After 3-4 days of inoculation, the local lesions number was then counted and recorded. The experiment was repeated three times with each tested sample. The inhibition rate of the tested samples was calculated according to the following formula (av denotes average and control was not treated with compounds):

(4) $$\begin{array}{c} \text{Inhibition}\;\text{rate}\;\%=\left[\frac{x-y}{x}\right]\times 100. \end{array}$$

Here, *x* = av is the number of local lesions in control, *y* = av is the number of local lesions in tested sample. 

*(3) Change in Chlorophyll Content of TMV Inoculated Tobacco Leaves after Treatment [24]*. The same age growing and healthy tobacco plant leaves were selected for viral tests. Foremost, the 7th leaf was selected and the whole leaf was inoculated with the virus followed by a simple water to rinse for surface sterilization. Leaves were dried and coated with virus inoculums for further analysis. Measurements were made on every second day for chlorophyll content. To determine chlorophyll content, the leaf samples were separated from plant and weighted accurately followed by putting it into 5 mL of 80% acetone at a temperature of 4°C. The dark extraction was performed overnight. The chlorophyll contents of tobacco leaves extracted over night are placed in a 1 cm thick cuvette with 80% of acetone as blank. The UV-Visible spectrometer was employed to measure absorbance of chlorophyll content at 645 nm and 663 nm to evaluate the Chl-a + Chl-b concentrations using the following formula.

Chl-a = 12.7A_663_ − 2.69A_645_; Chl-b = 22.7A_645_  −4.68A_663_.

Total chlorophyll content Chl-a + Chl-b = [20.2A_645_  ±  8.02A_663_]*V* × *W*/1000.

In the above formula, A_645_ and A_663_ are the absorbance values at the corresponding wave lengths, “*V*” is the volume of extraction and “*W*” is the weight of fresh leaf. Finally, chlorophyll content is expressed in mg·g^−1^FW.

#### 2.2.2. Antimicrobial Activity

*(1) Antibacterial Activity*. Disc diffusion method [25, 26] was employed to screen the significant antibacterial activity of the synthesized urea and thiourea derivatives **8**(**a**–**j**) against different bacteria such as *Streptococcus aureus* (ATCC-25923), *Pseudomonas aeruginosa* (ATCC-25619), and *Escherichia coli* (ATCC-9637). Ciprofloxacin was used as a standard drug for antibacterial studies. A standard inoculums of 1-2 × 10^−7^ c.f.u/mL (0.5 McFarland standards) was introduced on to the surface of sterile agar plates, and a sterile glass spreader was used for even distribution of the inoculums. Discs measuring 6 mm in diameter were prepared from Whatman No. 1 filter paper and sterilized by dry heat at 140°C for an hour. The dry sterilized discs soaked in a known concentration (200 *μ*g/mL) of the test compounds were placed in nutrient agar medium. Blank test showed that DMSO used in the preparation of the test solutions does not affect the bacteria. The plates after inoculation were inverted and incubated for 24 h at 37°C. The zone of inhibition around the disc which was calculated edge to edge zone of the confluent growth usually corresponds to the sharpest edge of the zone and was measured in millimeters. All tests were repeated three times and average data was taken as final result. Minimum inhibitory concentrations (MICs) were also determined by microbroth dilution technique [27–29]. Specifically 0.1 mL of standardized inoculum (1-2 × 10^7^ c.f.u/mL) was added to test tubes and incubated for 24 h at 37°C and two controls were maintained for each test sample. The growth was monitored visually and spectrophotometrically. The lowest concentration (highest dilution) required to arrest the growth of bacteria was regarded as minimum inhibitory concentration (MIC).

*(2) Antifungal Activity*. The antifungal activity of the titled urea and thiourea derivatives **8**(**a**–**j**) were screened against fungal strains such as *Aspergillus flavus* (MTCC-1884), *Aspergillus niger* (MTCC-1881), and *Candida albicans* (ATCC- 2091) using agar disc-diffusion method [27–29]. The fungal strains were maintained on Potato Dextrose Agar (PDA) medium (Hi-Media). A loop full of culture from the slant was inoculated into the Potato Dextrose broth and incubated at 37°C for 48–72 h. This culture (0.1 mL) was spread on the potato dextrose agar plate and a sterile glass spreader was used for even distribution of the inoculums. All the compounds were dissolved in dimethyl sulfoxide (DMSO). Sterile discs of Whatman No. 1 filter paper of about 6 mm diameter were dried and soaked in 200 *μ*g/mL concentration of the test samples. The soaked discs were impregnated on the surface of the media and incubated for 48–72 h at 37°C. Blank test showed that DMSO used in the preparations of the test solutions does not affect the test fungi. The zone of inhibition around the disc was the calculated edge to edge zone of the confluent growth which usually corresponds to the sharpest edge of the zone and was measured in millimeters. All tests were repeated three times and average data was taken as final result. Fluconazole was used as a standard drug for antifungal study. Minimum Inhibitory Concentration (MIC) of the tested samples was determined by micro-broth-dilution method [27–29]. Specifically 0.1 mL of standardized inoculum (1-2 × 10^7^ c.f.u/mL) was added to each test tube. The tubes were incubated aerobically at 37°C for 48–72 h. Control was maintained for each test sample. The lowest concentrations (highest dilution) of test compound that produced no visible signs of fungal growth (no turbidity) when compared with the control tubes were regarded as MICs.

## 3. Results and Discussion

### 3.1. Chemistry

**Scheme 1:**
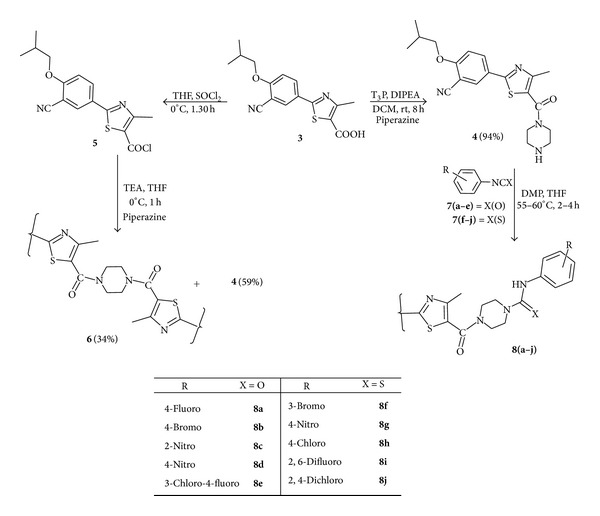
Synthesis of urea and thiourea derivatives of piperazine tagged with febuxostat.

The synthesis of urea **8**(**a**–**e**) and thiourea **8**(**f**–**j**) derivatives of piperazine tagged with febuxostat was accomplished. An intermediate, 2-isobutoxy-5-(4-methyl-2-(piperazine-1-carbonyl)thiazol-5-yl)benzonitrile (**4**) (Scheme 1) was synthesized according to two methods.

Primarily, we were attempting to synthesize key intermediate **4** by reacting piperazine with 5-(3-isobutoxyphenyl)-4-methylthiazole-2-carbonyl chloride (**5**), which was obtained by the conversion of carboxylic acid group in febuxostat (**3**) into acid chloride in the presence of thionyl chloride at 0°C. This reaction afforded 59% of the desired key intermediate **4 **along with 34% of dimerized product, 5,5′-(2,2′-(piperazine-1,4-diylbis(oxomethylene))bis(4-methylthiazole-5,2-diyl))bis(2-isobutoxybenzonitrile) (**6**). Also, the same model reaction was attempted at −15°C to improve the yield of the desired product **4**. Little enhancement in the yield of compound **4** (68%) was observed. Later, another method was adopted to prevent the formation of dimerized product; the carboxylic acid group in febuxostat was condensed with piperazine in DCM in the presence of T_3_P (propylphosphonic anhydride) at room temperature with vigorous stirring. The reaction provided a high yield of 94% desired key intermediate **4** with simple work-up procedure such as filtration and recrystallization from methanol. Finally, title urea and thiourea derivatives **8**(**a**–**j**) were achieved in high yields through the reaction of intermediate **4** with various pharmacologically active functionalized phenyl isocyanates **7**(**a**–**e**) and phenyl isothiocyanates **7**(**f**–**j**) in tetrahydrofuran (THF) using N, N-dimethylpiperazine as a base. It was also observed that isocyanates and isothiocyanates containing electron-withdrawing functionalities (**8c**, **8d**,** 8g**, and** 8i**) gave good yields in comparison with substrates bearing donating functional groups (**8b** and **8f**). The synthesized compounds were reported for the first time as per the literature knowledge. The physical characteristics of the title compounds are represented in Table 1.

The structures of the newly synthesized compounds **8**(**a**–**j**) were characterized by IR, ^1^H/^13^C NMR, mass spectral data, and elemental analysis. IR spectrum of compound **4** displayed strong absorption band at 3324 cm^−1^ for –NH (str) of piperazine, and the disappearance of the absorption band for –OH stretching of –COOH in febuxostat at 3418 cm^−1^ indicated the formation of intermediate compound, 2-isobutoxy-5-(4-methyl-2-(piperazine-1-carbonyl)thiazol-5-yl)benzonitrile (**4**). The appearance of the bands in all IR spectra of synthesized compounds in the region of 1720–1640 for (–C=O), and 1660–1590 (–C=S) indicated the formation of titled urea and thiourea derivatives **8**(**a**–**j**), respectively. In ^1^H-NMR spectrum of the compound **4**, the chemical shift at *δ*2.85 is assigned to –NH proton of intermediate **4**, and the disappearance of chemical shift value at *δ*11.3 of –COOH in febuxostat indicated the formation of intermediate **4**. Chemical shift values in the regions 8.15–10.98 ppm and 6.82–8.78 ppm are assigned to protons of –NH in urea/thiourea derivatives and aromatic protons, respectively. ^13^C-NMR spectra gave signals in the regions 154.4–168.3 ppm and 158.4–179.9 ppm for –C=O and–C=S, respectively. The appearance of the molecular ion peaks in the mass spectra and the correlated composition of tested compounds in elemental analysis with calculated composition have given further evidence for the characterization of the synthesized compounds. The spectral data of titled compounds are presented in the experimental part.

### 3.2. Biological Activity

**Figure 2:**
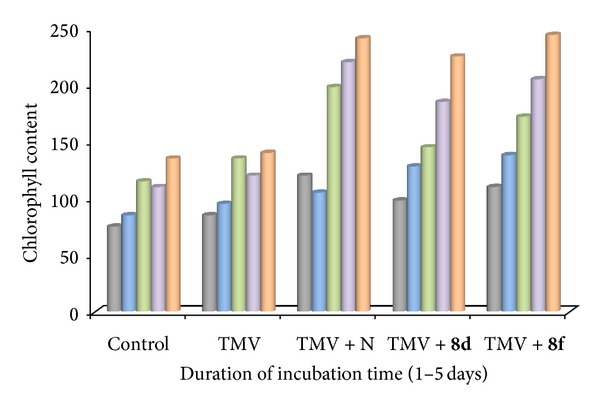
Change in the chlorophyll content in the tobacco leaves after treatment with **8d** and **8f**.

Antiviral activity of title compounds **8**(**a**–**j**) was screened against *Tobacco mosaic virus* and the results are given in Tables 2 and 3 and Figure 2. The results of *in vivo* inactivation rate, protective effect, and curative effects of the urea and thiourea derivatives **8**(**a**–**j**) against TMV are presented in Table 2. All the title compounds exhibited potent to moderate inhibition activity against TMV. The 4-nitrophenyl urea substituted compound **8d** and 3-bromophenyl thiourea substituted compound **8f** showed high inactivation of infection, that is, inhibition rate of **8d** and **8f **are 85.72 ± 0.33 and 85.93 ± 0.54, respectively. In *in vivo*, for **8d** and **8f**, infection protective effects are 58.72 ± 1.18 and 58.29 ± 0.76 and infection curative effects are 57.34 ± 0.82 and 56.31 ± 0.97, respectively. The Extent of viral infection in plants leads to proliferation expansion and destruction of the chloroplast, and these factors retarding the production of chlorophyll, subsequently causing leaf chlorosis and mosaic. It reduces the photosynthetic pigment in TMV infected leaves. The host's resistance of TMV infected leaves is enhanced when these leaves were treated with antiviral agents. Furthermore, the efficacy of the potent titled compounds **8d**, **8f** on the chlorophyll content in tobacco was examined and results are tabulated in Table 3 and Figure 2. The biological data revealed that the chlorophyll content in virus inoculated tobacco leaves was increased during 1–5 days and reached the highest value on the 5th day when these leaves are sprayed with potent synthesized compounds **8d** and **8f**. Among them, compound **8f** exhibited better effectiveness and **8d** showed less potency when compared with the standard, Nignanmycin. The compounds **8d** and **8f** significantly enhanced the chlorophyll content, indicating the demolish of virus of the tobacco and enhancing hosts resistance towards diseases.

The title compounds were screened against *S. aureus*, *P. aeruginosa*, and *E. coli* bacterial strains and against *A. flavus*, *A. niger*, and *C. albicans* fungal strains using disc-diffusion method. The minimum inhibitory concentration of the tested samples was also examined and the results are presented in Table 4. Groups like 4-fluorophenyl in **8a**, 4-nitrophenyl in **8d** of urea derivatives, and 3-bromophenyl in **8f**, 2,4-dichlorophenyl in **8j** of thiourea derivatives might be responsible for potent antimicrobial activity. Compound **8e** exhibited potent activity against *P. aeruginosa*. Compound **8f **has the lowest MIC value (15) against *S*. *aureus*.

All the compounds exhibited potent to moderate inhibitory activities against viral, bacterial, and fungal organisms. Especially the compounds **8a**, **8d**, **8f**, and **8j** exhibited good results against the above organisms at lower concentration when compared with other compounds.

## 4. Conclusion

In conclusion, a series of new urea and thiourea derivatives of piperazine tagged with febuxostat drug was accomplished in high yields. In the development of the target molecules, the piperazine is effectively coupled with febuxostat in high yields in the presence of T_3_P reagent. The antiviral activity against TMV and antimicrobial activity of the titled compounds were evaluated. The compounds **8d** (4-nitrophenyl urea substituted derivative) and **8f** (3-bromophenyl thiourea substituted derivative) were found to be promising antiviral agents and compounds **8a** (4-fluorophenyl urea substituted derivative) and **8j** (2, 4-dichlorophenyl thiourea substituted derivative) exhibited good antimicrobial activity. With little exception, overall, it was observed that the titled compounds meritoriously worked as antiviral agents.

## Figures and Tables

**Table 1:** Physical data of titled urea and thiourea derivatives **8(a–j)**.

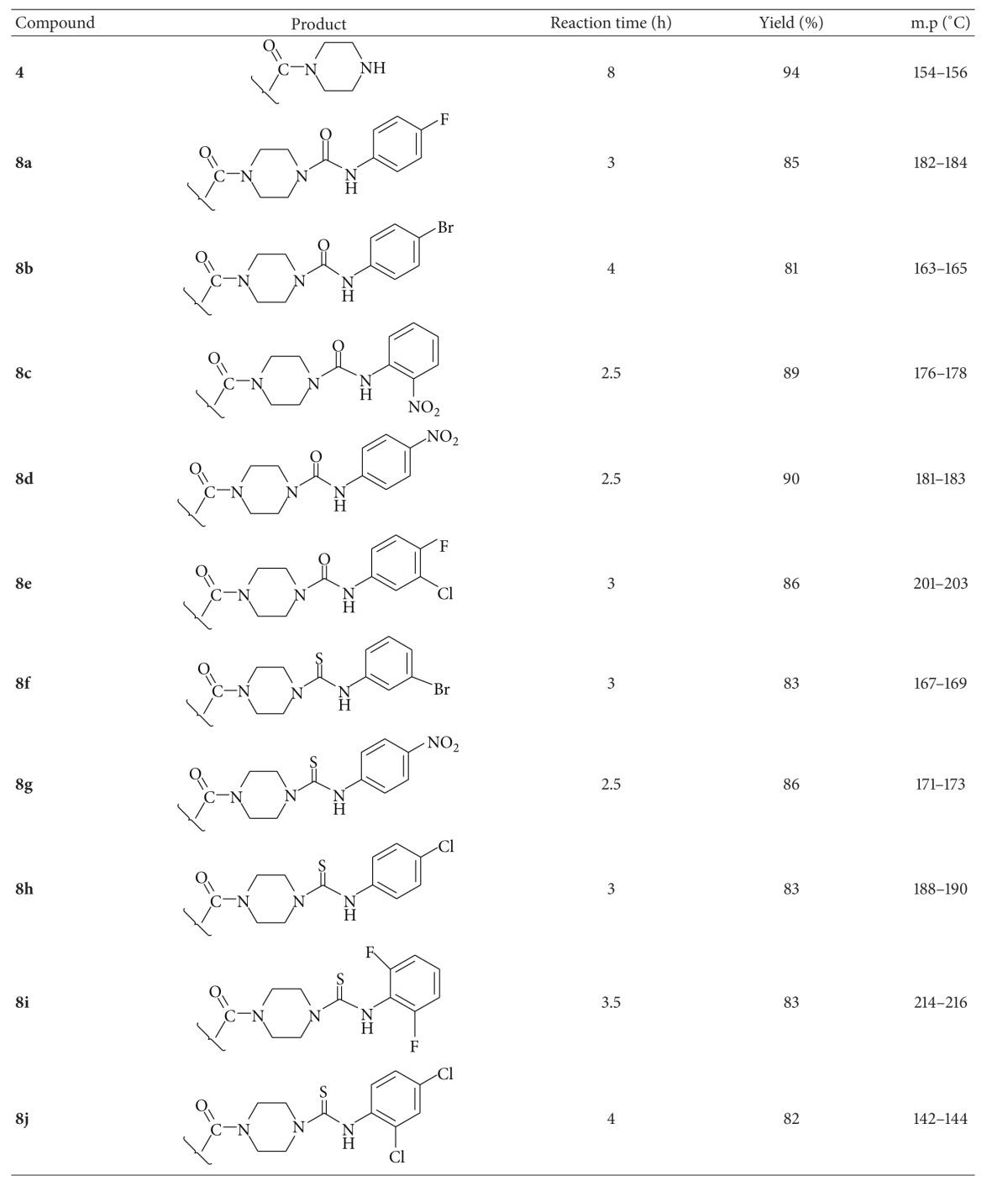

**Table 2:** The inhibitory activity of the titled urea and thiourea derivatives **8(a–j)** on TMV.

Compound^a^	Inactivation rate (%) ± SD	Protective effect (%) ± SD	Curative effect (%) ± SD
**4**	57.54 ± 0.18	39.82 ± 0.97	36.02 ± 0.72
**8a**	74.41 ± 0.56	41.24 ± 0.76	52.73 ± 1.43
**8b**	72.55 ± 0.74	40.57 ± 1.28	49.75 ± 0.76
**8c**	78.43 ± 1.01	52.31 ± 0.84	51.28 ± 0.69
**8d**	85.72 ± 0.33	58.72 ± 1.18	57.34 ± 0.82
**8e**	65.28 ± 0.97	39.86 ± 0.49	49.28 ± 0.86
**8f**	85.93 ± 0.54	58.29 ± 0.76	56.31 ± 0.97
**8g**	64.56 ± 1.27	45.53 ± 0.86	48.29 ± 1.08
**8h**	81.33 ± 0.79	58.88 ± 0.78	56.03 ± 1.10
**8i**	72.74 ± 0.48	50.73 ± 1.25	42.49 ± 0.92
**8j**	63.26 ± 0.98	47.96 ± 0.44	46.23 ± 0.75
Stand	88.45 ± 0.68	59.62 ± 0.74	57.27 ± 0.54

^a^The title compounds tested at 500 *µ*g/L.

Stand: Ningnanmycin, SD: standard deviation.

**Table 3:** Change in chlorophyll content (mg·g^−1^ FW) in the tobacco leaves after treatment with **8d** and **8f**.

Duration of incubation^b^ (days)	Control	TMV	TMV + N	TMV + **8d**	TMV + **8f**
1	75	85	120	98	110
2	85	95	105	128	138
3	115	135	198	145	172
4	110	120	220	185	205
5	135	140	241	225	244

^b^Incubation period in days, TMV: *Tobacco mosaic virus*, N: Ningnanmycin.

**Table 4:** Antimicrobial activity of the titled urea and thiourea derivatives **8(a–j)**.

Compd.	Bacterial culture						Fungal culture					
	*S. aureus *		*P. aeruginosa *		*E. coli *		*A. flavus *		*A. niger *		*C. albicans *	
	ZOI^c^	MIC	ZOI^a^	MIC	ZOI^a^	MIC	ZOI^a^	MIC	ZOI^a^	MIC	ZOI^a^	MIC
**4**	14.9	60	13.4	65	12.7	50	10.9	65	10.5	65	11.3	45
**8a**	20.8	17	19.9	20	23.8	17	18.6	30	19.3	30	18.8	35
**8b**	16.3	40	19.2	30	17.3	45	13.8	80	13.7	80	16.3	40
**8c**	17.1	18	18.5	45	17.6	55	14.3	80	14.4	75	14.5	55
**8d**	19.8	20	20.5	20	24.7	15	19.9	20	19.6	30	19.8	20
**8e**	18.9	30	20.8	20	19.4	35	17.5	45	16.8	65	17.7	30
**8f**	20.1	15	19.8	25	20.5	30	19.2	20	20.1	20	19.3	25
**8g**	17.8	45	17.3	35	18.3	70	16.4	50	17.8	40	16.4	30
**8h**	16.1	40	16.2	35	15.2	70	13.9	80	14.5	70	13.8	80
**8i**	16.7	30	18.8	40	17.9	45	16.1	45	18.3	35	15.9	70
**8j**	19.7	25	20.8	20	22.8	20	19.0	25	19.6	25	19.7	20
Ciprofloxacin	22	04	23	05	27	04	—	—	—	—	—	—
Fluconazole	—	—	—	—	—	—	21	04	23	04	22	05

ZOI: zone of inhibition in mm.

^c^Concentration of compounds at 200 *µ*g/mL.
